# PLANiTS: a curated sequence reference dataset for plant ITS DNA metabarcoding

**DOI:** 10.1093/database/baz155

**Published:** 2020-02-04

**Authors:** Elisa Banchi, Claudio G Ametrano, Samuele Greco, David Stanković, Lucia Muggia, Alberto Pallavicini

**Affiliations:** 1 Department of Life Sciences, University of Trieste, via Giorgieri 5, 34127, Trieste, Italy; 2 Division of Oceanography, National Institute of Oceanography and Applied Geophysics, via Piccard 54, 34151, Trieste, Italy; 3 Marine Biology Station, National Institute of Biology, Fornače 41, Piran, Slovenia; 4 Department of Biology and Evoliution of Marine Organisms, Stazione Zoologica Anton Dohrn, Villa Comunale, 80121 Naples, Italy

## Abstract

DNA metabarcoding combines DNA barcoding with high-throughput sequencing to identify different taxa within environmental communities. The ITS has already been proposed and widely used as universal barcode marker for plants, but a comprehensive, updated and accurate reference dataset of plant ITS sequences has not been available so far. Here, we constructed reference datasets of Viridiplantae ITS1, ITS2 and entire ITS sequences including both Chlorophyta and Streptophyta. The sequences were retrieved from NCBI, and the ITS region was extracted. The sequences underwent identity check to remove misidentified records and were clustered at 99% identity to reduce redundancy and computational effort. For this step, we developed a script called ‘better clustering for QIIME’ (bc4q) to ensure that the representative sequences are chosen according to the composition of the cluster at a different taxonomic level. The three datasets obtained with the bc4q script are PLANiTS1 (100 224 sequences), PLANiTS2 (96 771 sequences) and PLANiTS (97 550 sequences), and all are pre-formatted for QIIME, being this the most used bioinformatic pipeline for metabarcoding analysis. Being curated and updated reference databases, PLANiTS1, PLANiTS2 and PLANiTS are proposed as a reliable, pivotal first step for a general standardization of plant DNA metabarcoding studies. The bc4q script is presented as a new tool useful in each research dealing with sequences clustering.

Database URL: https://github.com/apallavicini/bc4q; https://github.com/apallavicini/PLANiTS.

## Introduction

DNA metabarcoding allows the automated identification of species present in an environmental sample (1) combining DNA barcoding and high-throughput sequencing techniques. Nowadays, DNA metabarcoding has found a broad range of applications, representing a key tool for understanding evolutionary history, functions and ecological diversity of organismal communities (2). It has also been applied for plant identification in different fields, such as palynology (3), melissopalynology (4), plant–pollinator interactions (5–7), identification of allergens (8, 9), environmental monitoring (10), dietary analyses (11, 12) and composition of herbal products (13).

Though many biases can be introduced by sampling strategy and sample processing, primer design, sequencing errors and bioinformatics pipelines (14), the accuracy and reproducibility of DNA metabarcoding heavily depend on the availability of a comprehensive, updated, accurate and standardized reference dataset for the targeted DNA barcode (5).

Sequence datasets of the nuclear ribosomal internal transcribed spacer (ITS rRNA; UNITE for fungi, 15, and ITSoneDB for eukaryotes, 16), the small (16S rRNA) and the large subunit ribosomal RNA (23S rRNA) for prokaryotes (RDP, 17; Greengenes, 18; SILVA, 19), the small (18S rRNA) and the large subunit ribosomal RNA (28S rRNA) for eukaryotes (SILVA, 19) and cytochrome oxidase subunit I (COI) for metazoans (CO1 Classifier, 20) have already been established. On the contrary, an acknowledged reference dataset specifically dedicated to the kingdom Planta is still missing, likely due to the deficiency of a robust, effective and largely accepted DNA barcode for plants. In this context, the determination of an official plant DNA barcode is still debated; indeed, different markers (either used individually or combined) have been proposed among several plastidial (e.g. rbcL and matK) and nuclear (e.g. ITS) loci (21).

However, multiple pieces of evidence support the suitability of the ITS region, ITS2 in particular, as the preferential marker for DNA metabarcoding in plants. This is mostly due to high levels of interspecific divergence and thus discriminatory power, which are higher than in short plastidial barcodes; further, it presents fewer amplification and sequencing problems (22). While both ITS1 and ITS2 subregions have been tested successfully over a broad range of plant taxa (23–25), the recent development of long read sequencing technologies such as MinION (Oxford Nanopore Technologies) or SMRT sequencing (Pacific Biosciences) enables the coverage of the whole ITS region. We expect that in the next years the discriminatory power of this barcode will be exploited for plant studies, as it has already been demonstrated for fungi (26).

National plant datasets have been established, such as the Great Britain database of national native plants and grasses (27), which has been recently used for DNA metabarcoding analyses (9). Other datasets regard specific geographic or ecologically characterized areas of interest, such as the one prepared for tropical herbal plants (28). Even though these datasets are of high quality, these resources are limited to only a small fraction of the whole plant biodiversity. This is also the case of other, global resources, such as the Barcode of Life Data system (BOLD; 29), which considers so far only about the 20% of the formally described land plants (30).

Sickel *et al*. (31) integrated the global ‘ITS2 database’ in the bioinformatic pipeline (32) when performing a study of honeybee-collected pollen using DNA metabarcoding. However, this database was updated in 2015 for the last time and held mainly information on the secondary structure of the ITS2 region but it does not include any identity check of the sequences. Recently, a rbcL reference dataset was developed by Bell *et al*. (5), as this marker, combined with the ITS2, aids in the identification at species level of some angiosperms.

At present, the large part of plant DNA metabarcoding studies rely on sequence data stored at and downloaded from NCBI (4, 8, 11, 12, 33) and sometimes complemented with own produced sequences (34). However, using the NCBI resources holds a grave disadvantage as the sequences are not checked for mistakes in taxonomy during deposition. Namely, a rather abundant number of sequences assigned to plant taxa but belonging to fungi can be frequently found in NCBI. These records usually belong to fungi that are present on or inside the plant tissue and that are sequenced instead of the target organism. To avoid taxonomic validation of each reference matching a sequence in a DNA metabarcoding survey, new bioinformatic pipelines that detect such taxonomic misidentifications automatically and new dedicated reference databases with a minimized number of misidentified sequences are needed.

In the frame of a DNA metabarcoding project aimed at analyzing plant and fungal diversity in aerobiological samples in Italy, we came across the problem of the lack of a reliable and comprehensive reference dataset for the ITS region of Viridiplanta. To overcome such issue, we provide a new script that checks whether the generated clusters in the reference database are composed mainly by one species and selects the representative sequence according to this. Furthermore, we generated a new, curated ITS reference dataset called ‘PLANiTS’ that we present here along with its innovative features.

## Materials and methods

Viridiplants ITS sequences were downloaded from NCBI on 11 April 2019 using the Entrez query ‘ITS’ OR ‘internal transcribed spacer’ OR ‘rRNA’ OR ‘ribosomal RNA’ AND ‘Viridiplantae’ [ORGN] AND ‘200: 4000’ [SLEN], to exclude genome contigs. In this way, we retrieved sequences of the ITS1 and the ITS2 fragments and complete the ITS region.

As one of the key points during the construction of a plant ITS database is the removal of sequences deposited in NCBI as plant sequences, but truly belonging to fungi, this study tackled this problem using two similarity-based methods, as follows:

(i) MEGAN (35) assigns sequences to taxa using the last common ancestor (LCA) algorithm and displays the inferred taxonomy. We used it to highlight sequences that, though having putatively the same taxonomy (based on the header of the fasta file), could not be aligned due to sequence divergence. In this case, the LCA algorithm cannot assign the reads at species level but places them into the ‘Not assigned’ or other higher taxonomic level, such as Eukarya. An ITS database with the downloaded sequences was firstly formatted using the ‘build’ tool from MALT 0.4.1 package (36) using the default parameters and 100% identity. Secondly the ‘run’ tool was used to run the formatted database against DNA metabarcoding samples that contained plant sequences. In this case, we used as test some aerobiological samples coming from our DNA metabarcoding study (Banchi *et al*. under review; see Results and discussion section) that presented a wide range of plant taxa and were suitable to this purpose. The produced rma files (compressed, indexed binary files that include sequence reads, alignments and taxonomic assignments) were imported in MEGAN 6 (35) for the benchmarking. The ‘Inspector’ tool provided in MEGAN was used to view the individual sequence comparisons upon which the assignment of a sequence to a taxon was based (35). Sequences suspected to be GenBank entries with a wrong taxonomic assignment were checked manually and further blasted in NCBI.

(ii) The BLAST command-line algorithm was used to blast the curated fungal UNITE database (15) against the downloaded ITS sequences to detect any positive match, potentially belonging to fungi. First, a database with sequences downloaded from NCBI was constructed using the ‘makeblastdb’ command, and then the UNITE database was blasted against it with the ‘blastn’ command at default parameters. The sequences that had a similarity hit were checked in NCBI.

As a result of the control procedures, several taxonomically misidentified fungal sequences were detected and discarded.

Sequences that presented the words ‘predicted’, ‘putative’, ‘uncharacterized’, ‘unverified’, ‘scaffold’, ‘protein’ and ‘hypothetical’ in the definition field (fasta header) were removed within the CLC Genomics Workbench v. 12 (Qiagen) software suite.

ITSx (37) was then used to extract ITS1, 5.8 and ITS2 fragments from the GenBank sequences.

To reconstruct the complete ITS region, the sequences that presented ITS1, 5.8 and ITS2 regions after ITSx were concatenated to obtain the complete region.

For each sequence, taxonomy was added at Phylum, Class, Order, Family, Genus and Species level with entrez_qiime (NA: not assigned; 38) and manually edited when needed.

**Figure 1:**
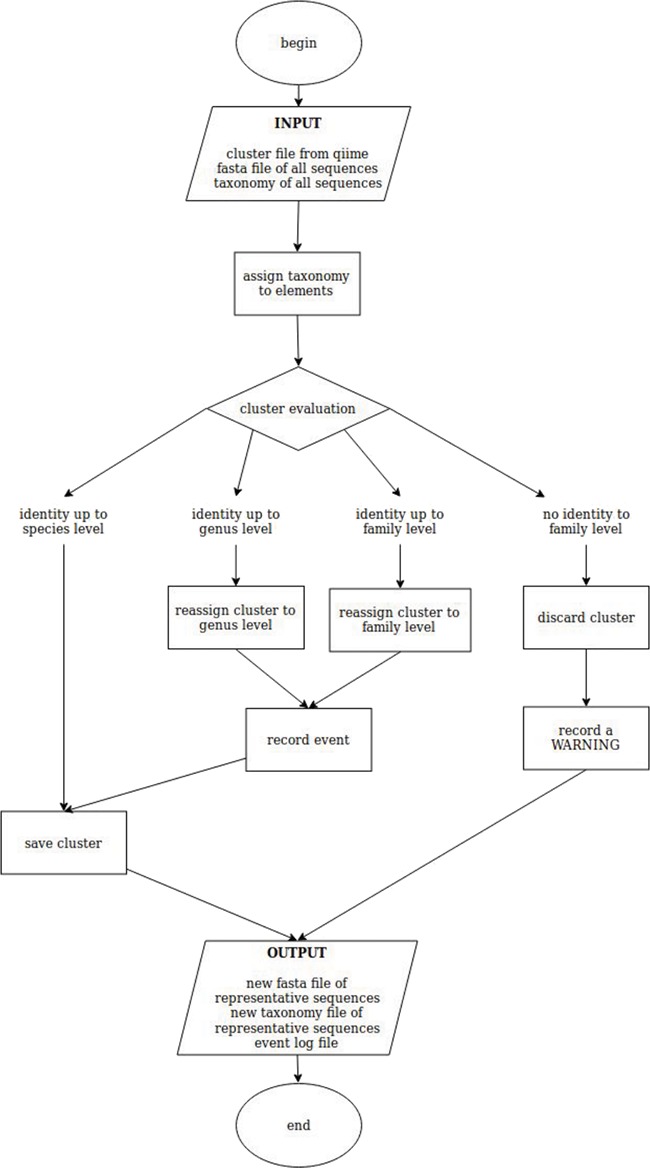
Schematic workflow of bc4q script for the evaluation of cd-hit clusters. The identity level is set at 90%.

To reduce redundancy and computational effort, the sequences were clustered with CD-HIT (39) at 99% identity. CD-HIT uses a greedy incremental clustering algorithm method and selects the longest sequence as representative of a certain cluster. Even if this approach is extremely fast and commonly accepted to make non redundant databases, it is prone to errors (i) if the longest sequence of a cluster has been erroneously identified while the others were corrected, or (ii) if the DNA marker has not enough discriminating power to resolve a certain taxon (e.g. at species level) and therefore a species is selected among others correctly represented in the cluster. To overcome these biases, we have developed a script called ‘better clusters for QIIME’ (bc4q) that checks whether the generated clusters are composed mainly by one species (more than 90% of the members of each cluster) and ensures that the representative sequence of each cluster is the most frequent one, by eventually replacing it with the one showing the highest number of counts. If no defined main species can be found in a cluster, the check is repeated at the genus level, and in case of no clear genus, at family level, re-ranking the members of the clusters to ensure that the most frequent genus (or family) is picked. If the genus (or family) check succeeds for a cluster, the cluster is kept, and the species (or the genus) is saved as ‘NA’ (not assigned) in the output file for taxonomy association. If the check fails also at the family level, the cluster is discarded, and the event is reported to the log together with the taxonomic information of the members of the failed cluster for manual review (Figure 1; code available at https://github.com/apallavicini/bc4q).

After a first run of the script, we manually checked those ‘warning’ clusters to identify misidentified plant accessions in ITS1, ITS2 and total ITS databases. These misidentified accessions were removed with CLC Genomics Workbench v. 12 (Qiagen) from the un-clustered databases that were clustered again with CD-HIT (39) at 99% identity. The script was re-run on the cleaned clusters. At the end, we produced three curated datasets which were named PLANiTS, PLANiTS1 and PLANiTS2, respectively, and are freely available (https://github.com/apallavicini/PLANiTS) in QIIME format, being QIIME the most common tool to analyze metabarcoding data (39). Moreover, the same format is required in the Microbial Genomics module of CLC Genomics Workbench v.12 (Qiagen). We are committed to updating the datasets with the newest GenBank sequences every 6 months to 1 year.

To further validate the performance of PLANiTS in plant taxonomic assignment, we compared its performance with the ITS2 Database (32) on the same dataset. From the ITS2 Database (32; http://its2.bioapps.biozentrum.uni-wuerzburg.de/), we downloaded the Viridiplantae sequences (consisting of 114 733 sequences) and we formatted them in QIIME format.

The PLANiTS database was not tested because the entire ITS fragment (up to 1500 bp) is still not sequenced by the most used DNA metabarcoding sequencing technologies used so far (e.g. Illumina, IonTorrent), which can read only up to ~400 bp. However, this database will be useful for long-read sequencing technologies that will become more common in the next years. The PLANiTS1 database was created because of the research that we were contemporaneously conducted on aerobiological samples, which induced us to generate the PLANiTS databases, as described in the Results and discussion section. In this study now under review, indeed, only the ITS2 was amplified, being it the most common marker in plant and fungal DNA barcoding and metabarcoding studies (21, 25) also in aerobiological field (14). Therefore, we principally chose to test PLANiTS2.

## Results and discussion

The query inserted in NCBI allows the export of 699 968 entries. These sequences were reduced to 446 025 after the first cleaning rounds (with MEGAN and with UNITE). A total of 592 taxonomic misidentifications were excluded because they consisted of sequences that were submitted to GenBank with plant names but instead represented fungal strains, such as *Cladosporium* (~13%), *Alternaria* (~7%), *Aspergillus* (~5%), *Aureobasidium* (~5%), *Fusarium* (~5%) and *Phoma* (~5%).

As NCBI does not double check the uploaded sequences, manual curation is needed to prevent the inclusion of sequences that may lead to incorrect taxonomic assignment and propagation of misidentifications (30). This issue is of particular importance for plants, as endophytic fungi are commonly amplified instead of—or co-amplified with—their targeted plant host. Fungi are known components of the plant microbiota present superficially or inside plant tissues as parasites, endophytes, pathogens and symptomless infections (41). It is therefore not surprising that the majority of the misidentified sequences corresponded to filamentous fungi, mould and yeast, which are widely distributed either as plant pathogens or endophytes (*Alternaria* and *Aureobasidium*) or are common in environments, such as soil and air (*Cladosporium*, *Aspergillus*, *Fusarium*, *Phoma*). Many of these sequences were erroneously included as Viridiplantae in other databases, such as the ITS2 Database of Ankebrand *et al*. (32).

In DNA metabarcoding studies, even if plant-specific primers are used, the potential co-amplification with other organisms is difficult to prevent (21); alternatively, in some studies it might be desirable to amplify plants and fungi simultaneously, thus universal primers are preferred. In either of these situations, the availability of a validated dataset is crucial.

ITSx extracted 289 408 ITS1 sequences, 267 752 5.8S sequences and 313 175 ITS2 sequences. ITS1 and ITS2 were trimmed in CLC Genomics Workbench v. 12 (Qiagen) to remove sequences shorter than 150 bp, resulting in 280 756 ITS1 and 306 345 ITS2 sequences, respectively. The 5.8S region was always complete, as sequenced between ITS1 and ITS2. The accessions that presented the three markers were then concatenated to produce the ITS reference, consisting of 267 752 ITS sequences.

Clustering with cd-hit at 99% produced 100 403 ITS1, 96 798 ITS2 and 96 190 entire ITS sequences (each corresponding to a cluster), respectively.

After the analysis with the bc4q script, 16 154 ITS1, 17 301 ITS2 and 15 729 whole ITS clusters were re-assigned at genus level as they contained more than one species. Moreover, 1529 ITS1, 1894 ITS2 and 1380 ITS clusters were highlighted as they contain more than two species. After the manual check, a total of 1558 accessions were considered as misidentified and were removed from the datasets.

After all the cleaning and clustering steps we obtained three curated final datasets (Table 1): PLANiTS1 (100 224 sequences), PLANiTS2 (96 771 sequences) and PLANiTS (97 550 sequences).

**Table 1:** Taxa number in the PLANiTS reference datasets

	**PLANiTS1**	**PLANiTS2**	**PLANiTS**
Phylum	2	2	2
Class	22	22	22
Order	133	134	130
Family	538	567	527
Genus	9927	10 055	9737
Species	57 324	58 893	55 690
Total seq.	100 224	96 771	97 550

Total number of sequences (Total seq.) is also reported

Among the total 168 viridiplant orders, 130–134 (~80%) are present in the reference datasets (Table 2). During the compilation of this reference dataset, we noticed that some classes are under-represented for the ITS barcode (i.e. the hornworts Anthocerotopsida, the liverwort Marchantiopsida and the fern Polypodiopsida). An increased sequencing effort in these groups would lead to a substantial improvement of the representation of the plant kingdom, which would lead to more complete and reliable taxonomic results in DNA barcoding and metabarcoding studies.

**Table 2:** List of plants orders

Chlorophyta
Chlorodendrophyceae
**Chlorodendrales**: 14 (4); 12 (4); 18 (3)
Chlorophyceae
**Chaetopeltidales**: 2 (2); 2 (2); 5 (4)
**Chaetophorales**: 23 (18); 23 (16); 41 (33)
**Chlamydomonadales**: 550 (345); 578 (376); 541 (339)
**Chlorosarcinales**: 11 (2); 8 (2); 17 (1)
**Oedogoniales**: 35 (18); 32 (16); 32 (17)
Phaeophilales
**Protosiphonales**: 3 (1); 4 (2); 5 (2)
**Sphaeropleales**: 393 (252); 389 (239); 401 (252)
**Tetrasporales:** 1 (1); 1 (1); 2 (2)
Chloropicophyceae
**Chloropicales**: 20 (15); 16 (12); 16 (10)
Mamiellophyceae
**Dolichomastigales**: 1 (1); 1 (1); 2 (2).
**Mamiellales:** 40 (22); 43 (25); 52 (19).
**Monomastigales**: 2 (2); 2 (2); 3 (2).
Palmophyllophyceae
Palmophyllales
Prasinococcales^*^
Pedinophyceae
Marsupiomonadales^*^
**Pedinomonadales**: 1 (1); 1 (1); 1 (1);
Scourfieldiales
Pyramimonadophyceae
**Pyramimonadales**: − (−); 6 (5); − (−).
Trebouxiophyceae
**Chlorellales**: 282 (138); 304 (151); 252 (118);
Ctenocladales
**Microthamniales**: 12 (4); 14 (4); 11 (3).
**Prasiolales**: 18 (12); 19 (14); 19 (13).
**Trebouxiales**: 641 (253); 667 (215); 559 (163).
Ulvophyceae
**Bryopsidales**: 3 (3); − (−); 161 (152).
Chlorocystidales
**Cladophorales**: 94 (29); 111 (63); 78 (32).
Dasycladales
**Ignatiales**: 1 (1); 1 (1); 1 (1).
Oltmansiellopsidales
Scotinosphaerales
**Trentepohliales**: 70 (27); 135 (48); 68 (28).
**Ulotrichales**: 44 (29); 36 (24); 41 (29).
**Ulvales**: 241 (179); 262 (195); 207 (136).
Streptophyta
Anthocerotopsida
Anthocerotales
**Dendrocerotales**:18 (12); 19 (13); 31 (21).
Notothyladales
Phymatocerotales
Bryopsida
Archidiales
**Bartramiales**: 64 (63); 63 (60); 65 (57).
**Bryales**: 92 (84); 80 (74); 172 (152).
**Bryoxiphiales**: 7 (7); 7 (7); 13 (13).
Buxbaumiales
**Dicranales**: 334 (307); 349 (325); 341 (296).
Diphysciales
**Encalyptales**: 2 (2); 2 (2); 3 (3).
**Funariales**: 31 (31); 31 (30); 28 (27).
**Gigaspermales**:2 (2); 2 (2); 5 (5).
**Grimmiales**: 212 (192); 202 (191); 217 (177).
**Hedwigiales**: 23 (17); 25 (19); 23 (18).
**Hookeriales**: 153 (140); 156 (142); 143 (130).
**Hypnales**: 758 (637); 804 (683); 871 (719).
Hypnodendrales^**^
**Orthotrichales**: 12 (12); 11 (11); 57 (43).
**Pottiales**: 487 (454); 489 (455); 449 (403).
**Pseudoditrichales**: 9 (8); 10 (9); 37 (36).
**Ptychomniales**: 5 (5); 6 (6); 4 (4).
**Rhizogoniales**: 20 (20); 16 (15); 24 (21).
**Scouleriales**: 23 (22); 24 (24); 21 (20).
**Splachnales**: 5 (5); 4 (4); 44 (40).
**Timmiales**: − (−); 2 (2); − (−).
Charophyceae
**Charales**: 25 (22); 24 (21); 27 (25).
Cycadopsida
**Cycadales**: 186 (150); 178 (139); 139 (99).
Ginkgoopsida
**Ginkgoales**: 3 (2); 3 (2); 7 (6).
Gnetopsida
**Ephedrales**: 5 (5); 5 (5); 27 (15).
**Gnetales**: 38 (31); 59 (54); 43 (36).
Welwitschiales
Jungermanniopsida
**Fossombroniales**: 23 (18); 24 (19); 20 (14).
**Jungermanniales**: 851 (765); 854 (753);
**Metzgeriales**: 21 (21); 21 (21); 128 (110).
**Pallaviciniales**:13 (12); 13 (12); 36 (34).
**Pelliales**: 1 (1); 1 (1); 1 (1).
Pleuroziales
**Porellales**: 830 (791); 1170 (1100); 1041 (956).
Ptilidiales^**^
Klebsormidiophyceae
**Klebsormidiales**: 46 (23); 40 (20); 53 (29).
Liliopsida
**Acorales**: 17 (10); 13 (7); 11 (5).
**Alismatales**: 814 (602); 897 (680); 728 (560).
**Arecales**: 282 (169); 323 (309); 373 (345).
**Asparagales**: 8045 (6094); 8254 (6277); 7856 (5930).
**Commelinales**: 15 (8); 15 (9); 11 (6).
**Dioscoreales**: 67 (61); 86 (80); 65 (60).
**Liliales**: 943 (730); 867 (673); 891 (675).
**Pandanales**: 4 (3); 9 (7); 3 (3).
Petrosaviales
**Poales**: 6947 (5770); 6546 (5397); 6432 (5182).
**Zingiberales**: 1565 (1211); 1419 (1083); 1487 (1113).
Lycopodiopsida
**Isoetales**: 79 (63); 68 (47); 90 (64).
**Lycopodiales**: 2 (2); 3 (3); 5 (5).
**Selaginellales**: 46 (43); 50 (46); 61 (54).
Magnoliopsida
**Apiales**:2299 (1806); 2147 (1673); 2224 (1729).
**Asterales**: 8694 (7103); 8976 (7347); 8450 (6780).
**Caryophyllales**: 4263 (3494); 4516 (3723); 4242 (3392).
**Celastrales**: 483 (388); 510 (420); 477 (370).
**Crossosomatales**: 28 (23); 29 (25); 32 (23).
**Dipsacales**: 524 (418); 561 (452); 522 (229).
**Ericales** 4026 (3207); 4224 (3382); 3940 (3065).
**Fabales**: 6783 (5507); 6921 (5644); 6355 (5050).
**Fagales**: 860 (695); 817 (640); 780 (570).
**Gentianales**: 4439 (3570); 4583 (3695); 4404 (3437).
**Geraniales**: 341 (269); 347 (270); 349 (273).
**Lamiales**: 8345 (6882); 8374 (6917); 8241 (6646).
**Laurales**: 722 (568); 703 (545); 540 (385).
**Magnoliales**: 74 (65); 93 (73); 106 (85).
**Malvales**: 1332 (1112); 1411 (1177); 1286 (1054).
**Myrtales**: 2668 (2056); 2734 (2103); 2360 (1771).
**Nymphaeales**: 125 (94); 120 (93); 111 (84).
**Piperales**: 544 (409); 553 (422); 634 (496).
**Proteales**: 469 (389); 475 (391); 487 (394).
**Ranunculales**: 1897 (1543); 2032 (1663); 1861 (1449).
**Rosales**: 3583 (2954); 3467 (2782); 3465 (2752).
**Santalales**: 338 (309); 403 (367); 365 (330).
**Sapindales**: 2357 (1992); 2477 (2090); 2361 (1953).
**Saxifragales**: 1617 (1318); 1699 (1385); 1577 (1234).
**Solanales**: 2067 (1773); 2079 (1787); 1868 (1530).
°Marchantiopsida
Blasiales
Lunulariales
**Marchantiales**: 21 (21); 21 (21); 67 (65).
Neohodgsoniales
Sphaerocarpales
°Pinopsida
**Araucariales**: 4 (3); 26 (13); 83 (51).
**Cupressales**: 225 (186); 219 (176); 193 (153).
**Pinales**: 63 (53); 68 (61); 127 (95).
°Polypodiopsida
**Cyatheales**: 5 (5); 5 (5); 5 (5).
**Equisetales**: 2 (2); 7 (7); 2 (2).
Gleicheniales
Hymenophyllales
Marattiales
Ophioglossales
Osmundales
**Polypodiales**: 32 (24); 31 (23); 35 (26).
**Psilotales**: 31 (31); 26 (26); 33 (33).
**Salviniales**: 28 (25); 28 (26); 27 (23).
**Schizaeales**: 4 (4); 2 (2); 4 (3).
°Polytrichopsida
**Polytrichales**: 41 (41); 41 (41); 78 (76).
°Sphagnopsida
**Sphagnales**: 108 (60); 120 (70); 90 (48).
°Takakiopsida
**Takakiales**: 1 (1); 1 (1); 1 (1).
°Tetraphidopsida
Tetraphidales^**^
°Zygnemophyceae
**Desmidiales**: 48 (46); 61 (59); 53 (50).
**Zygnematales**: 2 (2); 2 (2); 2 (2).
°Class not assigned
**Aquifoliales**: 270 (230); 249 (207); 246 (211).
**Austrobaileyales**: 41 (27); 40 (22); 39 (22).
Berberidopsidales
**Boraginales**: 1103 (961); 1238 (1072); 1118 (965).
**Brassicales**: 2377 (1926); 2744 (2311); 2504 (2055).
**Bruniales**: 35 (33); 37 (35); 37 (34).
**Buxales**: 25 (22); 29 (25); 26 (21).
**Canellales**: 36 (27); 41 (32); 36 (31).
**Ceratophyllales**: 13 (12); 18 (16); 13 (11).
**Chloranthales**: 40 (33); 44 (39); 38 (31).
**Cornales**: 274 (226); 350 (290); 281 (229).
**Cucurbitales**: 1155 (976); 1139 (958); 1202 (1012).
**Dilleniales**: 59 (52); 53 (44); 45 (35).
Escalloniales
**Garryales**: 9 (5); 14 (6); 16 (10).
**Gunnerales**: 25 (19); 17 (11); 18 (12).
Huerteales^**^
**Icacinales**: 2 (2); 3 (3); 5 (3).
**Malpighiales**: 4680 (3892); 4880 (4094); 4707 (3861).
**Metteniusales**: 1 (1); 1 (1); 5 (5).
**Oxalidales**: 378 (313); 380 (315); 383 (300).
Paracryphiales
Picramniales
**Trochodendrales**: 18 (18); 19 (19); 4 (4).
Vahliales
**Vitales**: 213 (189); 167 (151); 208 (182).
**Zygophyllales**: 213 (189); 158 (145); 166 (151).

Orders in bold are the one present in PLANiTS reference dataset. *Orders present only in PLANiTS1, **orders present only in PLANiTS2. For each order the total number of sequences identified in the three databases (PLANiTS, PLANiTS1 and PLANiTS2) is reported, and the corresponding number of sequences identified at the species level is additionally reported in parentheses

In the three datasets, 15 691 ITS1, 16 949 ITS2 and 15 643 whole ITS clusters were re-assigned at genus level, while 1204 ITS1, 1338 ITS2 and 1049 ITS clusters were re-assigned at family level. Cluster re-assigned at family level mainly contain few recurrent species from the following plant families: Scenedesmaceae and Siphonocladaceae (Chlorophyceae, Chlorophyta), Brachytheciaceae (Bryopsida, Streptophyta), Lejeuneaceae (Jungermanniopsida, Streptophyta) and Araceae (Liliopsida, Streptophyta). Whether a genus or species identification is needed for these genera, we suggest improving the resolution of molecular identification by adding another barcode, such as matK.

The bc4q script can also serve as a new resource for the construction of other reference databases, as the clustering step is a crucial part of data management.

We have successfully tested PLANiTS2 reference database with an *ad hoc* mock community and 110 aerobiological samples collected with volumetric samplers in different sites of North and Central Italy from a three-season survey in which DNA metabarcoding was applied to study plant diversity (Banchi *et al.* under review). The same samples were also analyzed using ITS2 Database (32) in order to compare the performance of the two resources.

The mock community was composed by seven plant taxa: one species of Chlorophyta, i.e. *Trebouxia gelatinosa*, and six species of Streptophyta, i.e. the Gymnosperm *Taxus baccata* and the Angiosperms *Acer campestre*, *Campanula* sp., *Corylus avellana*, *Tulipa gesneriana* and *Wisteria* sp.

The plant samples were chosen among the plants available at the Botanic Garden of the University of Trieste with the aim to include the widest range of taxa. The pollen was collected timely in the seasons. Chlorophyta was chosen among the algal cultures stored at the University of Trieste, representing a cosmopolitan terrestrial algal genus.

DNA extraction, library preparation and sequencing were performed as described in Banchi *et al*. (42) using as forward primer the reverse complement of ITS-u2 and ITS-p4 as reverse primer (21). Taxonomic assignment was performed with QIIME2 (40) with the alignment-based taxonomy consensus method based on vsearch 2.0.3 (43) applying the 97% identity limit and PLANiTS2 or ITS2 Database (32) as reference.

The taxonomic composition of the mock community analyzed with PLANiTS2 allowed for correct assignment at the genus level. In particular, *C. avellana*, *A. campestre* and *T. gesneriana* were assigned at species level, while *T. gelatinosa*, *T. baccata* and *Wisteria* sp. were identified up to their genera.

Using the ITS2 Database (32), the green alga *Trebouxia* was not detected. *C. avellana*, *A. campestre* and *Taxus baccata* were assigned at species level, while *T. gesneriana* and *Wisteria* sp. were identified up to their genera. However, also other taxa were detected; *Dioscorea polystachya*, *Avena sativa*, *Fargesia fungosa*, *Oryza sativa*, *Pueraria montana* and *Pteris vittata*. These are misidentified and belong to fungal species: *Cladosporium* sp.*, Aureobasidium pullulans*, *Cladosporium* sp., *Cladosporium* sp., *Debaryomyces* sp*.* and *Epicoccum* sp., respectively.

The analysis of aerobiological samples using PLANiTS2 recovered 158 plant genera. Plant taxonomic composition was mostly influenced by season. *Corylus* was the most abundant genus recovered in spring, whereas in summer and autumn the highest abundance was detected for *Brassica* followed by *Linum*, *Cucurmis* and *Daucus* (Banchi *et al.* under review).

With ITS2 Database (32), the total number of genera (168) and the results for the most represented taxa across samples (*Corylus, Brassica*, *Linum*, *Campanula*, *Cucumis*, *Daucus*) are comparable between the two databases. However, also here fungal taxa misidentified as plant taxa, are present even in high rank position (i.e. 14th) and represent ~5% of the sequences. These are listed as *Pueraria*, *Dioscorea*, *Pteris* and *Fallopia*, belonging instead to *Debaryomyces* sp*.*, *Cladosporium* sp., *Epicoccum* sp. and *Filobasidium* sp.

These results show how the cleaning and the clustering of plant sequences are important for a reliable taxonomic assignment, especially in the analysis of mixed environmental samples, such as air samples, where plants and fungi are present.

PLANiTS1, PLANiTS2 and PLANiTS are curated, reliable and updated reference databases and we propose them as a pivotal first step for a general standardization of plant DNA metabarcoding studies, in the prospect of facilitating the comparison of data among different researches dealing with plant identification at deep taxonomic level.
